# Human papillomavirus prevalence, genotype distribution, risk factors, and cervical pathology association in women aged 50 years and older: a retrospective cross-sectional study in Xinjiang, China

**DOI:** 10.3389/fonc.2025.1694755

**Published:** 2026-01-12

**Authors:** Nan Li, Li He, Huabin Zhang, Gulimire Turson, Jiaqi Lou, Hongfang Cheng

**Affiliations:** 1 Department of Gynecology, People’s Hospital of Kuqa, Kuqa, China; 2 Department of Gynecology, Women and Children’s Hospital of Ningbo University, Ningbo, Zhejiang, China; 3 Burn Department, Ningbo No.2 Hospital, Ningbo, Zhejiang, China

**Keywords:** cervical pathology, genotype distribution, HPV infection, older women, risk factors, Xinjiang region

## Abstract

**Background:**

Persistent high-risk human papillomavirus (HPV) infection is the primary cause of cervical cancer, but epidemiological data on HPV in women aged ≥50 years—especially in ethnically diverse, resource-limited regions such as Xinjiang, China—remain insufficient. Older women face underrecognized risks due to age-related immune decline and inadequate screening, highlighting the need for targeted research.

**Methods:**

A retrospective cross-sectional study was conducted among 640 women aged ≥50 years who underwent cervical HPV testing at a tertiary hospital in Kuqa, Xinjiang, from January 2024 to March 2025. *A priori* sample size calculation indicated a minimum requirement of 544 participants, which was exceeded. Sociodemographic (age, education), behavioral (sexual activity frequency, smoking and alcohol use), and clinical variables (age at menopause, parity, history of cervical surgery, body mass index [BMI]) were extracted from medical records. HPV status and genotypes were detected using a PCR-based DNA microarray, and cervical pathology was classified according to WHO criteria. Statistical analyses were preceded by assessment of variable normality. Analyses included descriptive statistics, binary and ordinal logistic regression, chi-square tests, t-tests/ANOVA, and point-biserial correlation analysis (SPSS 26.0; p<0.05).

**Results:**

The overall HPV prevalence was 20.6% (132/640), with high-risk genotypes HPV16 (29.5%), HPV53 (18.2%), and HPV58 (12.1%) being the most prevalent. Binary logistic regression showed that a history of cervical surgery (e.g., conization) was strongly associated with a reduced likelihood of HPV positivity (OR=0.015, 95% CI: 0.008--0.028, p<0.001). Univariate analysis indicated that sexual activity frequency was higher in HPV-positive than HPV-negative women (2.35 ± 1.14 vs. 2.11 ± 1.10, t=2.225, p=0.026), but this association was not significant in the multivariate model (OR=1.020, 95% CI: 0.796--1.307, p=0.874). Point-biserial correlation analysis revealed a negative association between HPV positivity and BMI (r_pb=-0.088, p<0.05). HPV-positive women had a higher rate of cervical intraepithelial neoplasia (CIN) and squamous cell carcinoma (SCC) compared with HPV-negative women (31.1% vs. 1.2%, p<0.001). A *post hoc* power analysis for the cervical surgery–HPV association yielded a power >99.9%.

**Conclusion:**

Women aged ≥50 years in Xinjiang have a non-negligible HPV prevalence, with HPV16 and HPV53 as dominant genotypes. A history of cervical surgery was associated with a markedly lower likelihood of HPV detection in this cross-sectional analysis. The association between sexual activity frequency and HPV status was not consistent across analyses and requires cautious interpretation. HPV positivity was strongly correlated with cervical pathological lesions, emphasizing the urgency of tailored screening and prevention strategies for this population.

## 1 Introduction

Cervical cancer remains the fourth most common malignancy among women worldwide, posing a significant burden on global public health systems, particularly in low- and middle-income countries (1). Extensive epidemiological and molecular evidence has established that persistent infection with high-risk types of human papillomavirus (hrHPV) is the necessary cause of most cervical cancer cases (2). Although the majority of HPV infections in younger women are transient and cleared spontaneously within one to two years due to effective immune responses, women aged 50 years and older represent a distinct epidemiological subgroup facing elevated risks. Immunosenescence—the gradual deterioration of the immune system associated with aging—compromises the clearance of viral infections and facilitates the persistence of hrHPV (3), thereby increasing the cumulative risk of developing high-grade cervical lesions and invasive carcinoma (4). Compounding this biological vulnerability, many national cervical cancer screening programs, including those in China, primarily target women aged 25–49 years, often leading to the neglect of older women who fall outside the recommended screening age range (5–7). Consequently, a considerable number of older women with persistent HPV infections or progressive precancerous lesions remain undiagnosed until symptomatic, resulting in delayed treatment and poorer clinical outcomes (8).

The Xinjiang Uygur Autonomous Region in northwestern China is characterized by a multi-ethnic population, including Han, Uygur, Kazakh, Hui, and other ethnic groups, as well as substantial geographic isolation and socioeconomic disparities (9). Although previous studies (10, 11) have examined HPV epidemiology in the general female population of Xinjiang, there is a notable scarcity of research focusing specifically on older women. Existing studies in the region are often limited by small sample sizes or by a lack of integration between HPV genotype data and histopathological outcomes. Certain risk factors—such as a history of cervical procedures, including conization, loop electrosurgical excision procedure (LEEP), or hysterectomy—have been inconsistently associated with HPV infection dynamics. Some studies suggest that surgical intervention may increase susceptibility to new infections due to epithelial disruption (12, 13), whereas others propose that excision of pre-neoplastic tissue may reduce viral load and decrease the risk of recurrence (14). These conflicting findings underscore the need for more nuanced investigations in well-defined populations.

To address these research gaps, this study aimed to (1) determine the overall and type-specific prevalence of HPV infection among women aged 50 years and older in Kuqa, Xinjiang; (2) identify sociodemographic, behavioral, and clinical risk factors associated with HPV positivity; and (3) evaluate the association between HPV infection status and cervical pathological outcomes, including low- and high-grade squamous intraepithelial lesions (LSIL/HSIL) and invasive squamous cell carcinoma (SCC). By providing localized, population-specific evidence, our findings seek to inform the development of targeted preventive strategies and optimize cervical cancer screening protocols for older women in underserved regions.

## 2 Methods

### 2.1 Ethical considerations

This study was approved by the Institutional Review Board of Kuqa People’s Hospital (Approval No.2024-83). Patient identifiers (e.g., name and phone number) were anonymized during data extraction to protect privacy. Informed consent was waived due to the retrospective nature of the study.

### 2.2 Study design and population

This retrospective cross-sectional study included consecutive women aged ≥50 years who underwent cervical HPV testing and gynecological examination at the Department of Gynecology, Kuqa People’s Hospital (a tertiary hospital in Xinjiang), from January 2024 to March 2025. *A priori* sample size calculation was performed for the primary descriptive objective of estimating HPV prevalence. Assuming an expected prevalence of 15% based on regional data, a 95% confidence level, and a desired precision (margin of error) of ±3%, the minimum required sample size was calculated to be 544 participants using the formula for estimating a single proportion: n=*Z*^2^×*p*(1−*p*)/e². The target enrollment exceeded this number to ensure sufficient precision and allow for multivariate analyses. This study was conducted in accordance with the ethical principles of the Declaration of Helsinki (15). The research protocol was reviewed and approved by the Medical Ethics Committee of Kuqa People’s Hospital (approval no. 2024-83). During data collection, patient identifiers were anonymized, and informed consent was waived because of the retrospective design.

Exclusion criteria were as follows: (1) incomplete demographic or clinical records (e.g., missing HPV test results or surgical history); (2) history of hysterectomy, which eliminates cervical tissue required for HPV detection; and (3) concurrent malignant tumors of other organ systems. Five participants were excluded due to incomplete HPV test results (n=3) or missing key demographic data (n=2). After data cleaning—including completeness checks, outlier detection, and collinearity diagnostics—640 valid samples were included in the final analysis.

### 2.3 Data collection

Data were extracted from electronic medical records and standardized case report forms, including:

Sociodemographic Variables: Age (categorized as 50–60 years, 61–70 years, and >70 years); education level (secondary school or below, junior college, bachelor’s degree); residence (rural or urban, based on registered address).
Behavioral Variables: Sexual activity frequency (categorized as none, 2–3 times per 6 months, 1–3 times per month, and ≥1 time per week); number of lifetime sexual partners (continuous variable); smoking and alcohol use (binary: “yes” if ever smoked or regularly consumed alcohol, “no” otherwise).
Clinical Variables: Menopausal status (binary: “yes” if ≥12 months of amenorrhea, “no” otherwise); age at menopause (continuous variable); parity (number of live births, continuous variable); history of cervical surgery (binary: “yes” if the participant had ever undergone cone biopsy, cervical biopsy, or loop electrosurgical excision procedure [LEEP], “no” otherwise). Myomectomy was not considered a cervical surgery and was reclassified during data analysis. Body mass index (BMI) was categorized according to WHO standards as 18.5–24.9 (normal), 25.0–29.9 (overweight), and ≥30.0 (obese).
Outcome Variables: HPV status and genotype: Cervical exfoliated cells were collected using a cytobrush, and HPV detection was performed using a PCR-based DNA microarray (Yaneng Biotechnology, Shenzhen, China). The assay identifies 21 HPV genotypes, including 15 high-risk types (HPV16, 18, 31, 33, 35, 39, 45, 51, 52, 53, 56, 58, 59, 66, and 68) and 6 low-risk types (HPV6, 11, 42, 43, 44, and 81). “HPV-positive” was defined as detection of any HPV genotype. Cervical pathology: Cervical biopsy or cone specimens were stained with hematoxylin–eosin and independently diagnosed by two senior pathologists according to the WHO 2020 classification as chronic cervicitis, cervical intraepithelial neoplasia grade 1 (CIN1), cervical intraepithelial neoplasia grade 2 or higher (CIN2+), or squamous cell carcinoma (SCC).

### 2.4 Statistical analysis

Data were cleaned and preprocessed to ensure quality. Missing values were handled by case-wise exclusion, and continuous variables were screened for outliers. The normality of continuous variables (age, age at menopause, BMI, and parity) was assessed using the Shapiro–Wilk test. Results are summarized in **Supplementary Table S3**. Variables that did not significantly deviate from normality (p ≥ 0.05) are presented as mean ± standard deviation (SD), whereas variables with significant deviation (p < 0.05) are presented as median with interquartile range (IQR). This assessment guided the selection of parametric versus non-parametric tests. Collinearity was evaluated using the variance inflation factor (VIF) (16), with all VIF values <5, indicating no severe multicollinearity.

Descriptive statistics were used to summarize sociodemographic and clinical characteristics. For group comparisons, independent-samples t-tests were used for normally distributed continuous variables, whereas the Mann–Whitney U test was applied to non-normally distributed variables. Binary logistic regression analysis was performed to identify factors associated with HPV positivity. Variables with p < 0.1 in univariate analyses (results shown in **Supplementary Table S1**) were entered into the multivariate model. Reference categories for all categorical variables in regression analyses are explicitly indicated in the tables. Ordinal logistic regression was used to explore associations between independent variables and ordinal cervical pathological outcomes (e.g., progression from chronic cervicitis to squamous cell carcinoma) (17). The proportional odds assumption was assessed using the Brant test, and no significant violation was detected (χ^2^ = 4.82, p = 0.305). Point-biserial correlation analysis was applied to assess linear associations between HPV status (binary) and continuous variables that met the normality assumption. Categorical variables were compared using chi-square tests. A *post hoc* power analysis was conducted for the primary association of interest (history of cervical surgery and HPV positivity) using G*Power 3.1, based on the observed effect size (OR=0.015), sample size (N=640), and an alpha level of 0.05. All analyses were performed using SPSS 26.0 (IBM Corp., Armonk, NY, USA), and a two-tailed p < 0.05 was considered statistically significant (18).

## 3 Results

### 3.1 Sample characteristics

**Figure 1:**
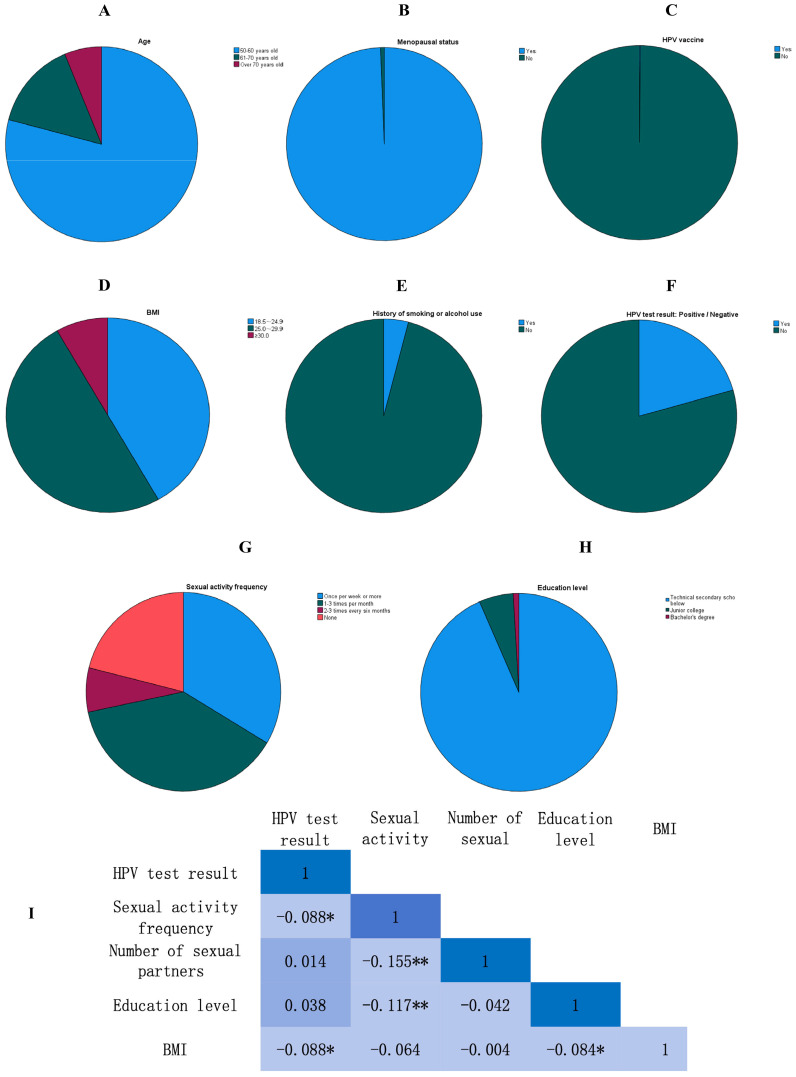
Sociodemographic, clinical, and key study outcome distributions among women aged ≥50 years in Kuqa, Xinjiang (n=640). **(A)** Age distribution of study participants; most women (79.1%) were aged 50–60 years. **(B)** Menopausal status; the majority of participants (95.8%) were postmenopausal. **(C)** HPV vaccination status; HPV vaccine coverage was extremely low (0.2%). **(D)** Distribution of body mass index (BMI) categories according to WHO standards; half of the participants (50.0%) were classified as overweight. **(E)** History of smoking or alcohol use; only a small proportion of women (4.1%) reported ever smoking or consuming alcohol regularly. **(F)** HPV test results; the overall HPV prevalence was 20.6% (132/640). **(G)** Sexual activity frequency distribution; the most common frequency was 1–3 times per month (38.1%). **(H)** Education level distribution; most participants (93.4%) had a secondary school education or below. **(I)** Heatmap of correlation coefficients among key variables. Color intensity represents the magnitude of Correlation coefficients are presented as Pearson’s r for continuous–continuous associations and point-biserial r_pb for binary–continuous associations (*p<0.05, **p<0.01). Variables include HPV test result (binary), sexual activity frequency (ordinal), number of lifetime sexual partners, education level (ordinal), and BMI (continuous). Warm colors indicate positive correlations, and cool colors indicate negative correlations. HPV, human papillomavirus; WHO, World Health Organization. Percentages are based on the total study sample (n=640).

A total of 640 women were included in the final analysis, with a mean age of 56.8 ± 7.2 years. Most participants (79.1%) were aged 50–60 years, followed by 14.7% aged 61–70 years and 6.3% aged >70 years. These age distributions, along with other key characteristics, are illustrated in **Figure 1**. The majority of women were postmenopausal (95.8%), with a mean age at menopause of 49.31 ± 2.11 years. Only 0.2% of participants had received HPV vaccination. Regarding BMI, 50.0% of women were classified as overweight, 8.3% as obese, and 41.7% as having normal BMI. Educational attainment was generally low, with 93.4% of participants having completed secondary school or less. Additionally, only 4.1% reported a history of smoking or alcohol use. Reliability analysis indicated acceptable internal consistency of the data collection scale (Cronbach’s α=0.746). Detailed sociodemographic and clinical characteristics are presented in **Table 1**. The internal consistency was calculated based on a set of sociodemographic and clinical variables extracted from medical records, including age group, education level, menopausal status, parity, BMI category, and behavioral factors (smoking/alcohol use, sexual activity frequency). Although these items were not derived from a standardized questionnaire, they formed a consistent data structure for reliability assessment in this retrospective context.

**Table 1:** Sociodemographic and clinical characteristics of the study population (n=640).

Variable	Category	Frequency (n)	Percentage (%)
Age	50--60 years	506	79.1
	61--70 years	94	14.7
	>70 years	40	6.3
HPV vaccination	Yes	1	0.2
	No	639	99.8
Menopausal status	Yes	613	95.8
	No	27	4.2
Sexual activity frequency	None*	135	21.1
	2--3 times/6 months	46	7.2
	1--3 times/month	244	38.1
	≥1 time/week	215	33.6
Smoking/alcohol use	Yes	26	4.1
	No	614	95.9
BMI	18.5--24.9 (normal)	267	41.7
	25.0--29.9 (overweight)	320	50.0
	≥30.0 (obese)	53	8.3
Education level	Secondary or below*	598	93.4
	Junior college	36	5.6
	Bachelor's degree	6	0.9
Cervical surgery history	Yes	89	13.9
	No*	551	86.1

*Denotes the reference category for variables included in regression analyses.

### 3.2 HPV prevalence and genotype distribution

The overall HPV prevalence was 20.6% (132/640). Among HPV-positive women, 87.9% (116/132) were infected with high-risk HPV genotypes, whereas 12.1% (16/132) had low-risk HPV infections. Genotype analysis showed that HPV16 was the most prevalent type (29.5%), followed by HPV53 (18.2%) and HPV58 (12.1%) (**Table 2**). Co-infection with multiple HPV genotypes was observed in 28.8% (38/132) of HPV-positive women. The most common co-infection pattern was HPV16 and HPV53 (n=8).

**Table 2:** Distribution of HPV genotypes among HPV-positive women (n=132).

HPV type	Category	Frequency (n)	Percentage (%)
HPV16	High-risk	39	29.5
HPV53	High-risk	24	18.2
HPV58	High-risk	16	12.1
HPV52	High-risk	12	9.1
HPV39	High-risk	10	7.6
HPV68	High-risk	8	6.1
Others (18,31,33, etc.)	High-risk	17	12.9
Low-risk HPV (6,11,81)	Low-risk	16	12.1

### 3.3 Risk factors for HPV positivity

Univariate analysis identified history of cervical surgery and sexual activity frequency as factors associated with HPV positivity (p < 0.1). Specifically, cervical surgery history was associated with a significantly reduced likelihood of HPV positivity (OR=0.016, 95% CI: 0.009–0.030, p < 0.001), whereas sexual activity frequency was associated with an increased likelihood of HPV positivity (OR per category increase=1.183, 95% CI: 1.020–1.372, p=0.027). These results are presented in **Supplementary Table S1**. These Variables meeting the inclusion criterion were entered into the multivariate logistic regression model, which demonstrated good overall fit (McFadden R²=0.412; Nagelkerke R²=0.537). In the adjusted model, cervical surgery history remained strongly associated with a reduced likelihood of HPV positivity (OR=0.015, 95% CI: 0.008–0.028, p < 0.001). In contrast, sexual activity frequency was no longer significantly associated with HPV positivity after adjustment (OR=1.020, 95% CI: 0.796–1.307, p=0.874). Education level and parity also showed no significant associations with HPV status (p > 0.05). Detailed regression results are provided in **Table 3**. The hoc power analysis indicated that the study was exceptionally well powered (>99.9%) to detect the observed association between cervical surgery history and HPV positivity.

**Table 3:** Binary logistic regression analysis of risk factors associated with HPV positivity (n=640).

Variable	Category / unit	Coefficient (B)	SE	Wald χ²	p-value	OR (95% CI)
Cervical surgery history	Yes vs. No (Ref)	-4.194	0.319	172.609	<0.001	0.015 (0.008–0.028)
Sexual activity frequency	Per category increase*	0.020	0.126	0.025	0.874	1.020 (0.796–1.307)
Education level	Junior college vs. Secondary or below (Ref)	-0.533	0.533	0.999	0.318	0.587 (0.206–1.669)
	Bachelor's vs. Secondary or below (Ref)	-0.322	0.947	0.116	0.734	0.725 (0.113–4.640)
Parity	Per birth increase	-0.046	0.252	0.033	0.855	0.955 (0.583–1.564)
Intercept		6.508	1.008	41.657	<0.001	

McFadden R² = 0.412; Nagelkerke R² = 0.537; Dependent variable: HPV positivity. Ref = Reference category.

*Sexual activity frequency was coded ordinally (1=None, 2=2-3 times/6 months, 3=1-3 times/month, 4=≥1 time/week) and treated as a continuous predictor (per category increase).

An ordinal logistic regression analysis was performed to evaluate the association between cervical surgery history and pathological progression (ordinal outcome: chronic cervicitis, CIN1, CIN2+, and squamous cell carcinoma). The model was statistically significant (χ²=105.62, p < 0.001). The proportional odds ratio for more severe pathological diagnosis among women with a history of cervical surgery was 0.12 (95% CI: 0.06-0.24, p < 0.001), indicating lower odds of advanced pathology in this cross-sectional analysis. These results are summarized in **Supplementary Table S2**.

### 3.4 Pearson correlation analysis

Point-biserial correlation analysis was conducted to examine linear relationships between HPV status (binary) and selected continuous variables. Only variables meeting the normality assumption (**Supplementary Table S3**) were included. HPV positivity was negatively correlated with BMI (after square-root transformation to approximate normality) (r_pb=-0.088, p < 0.05). The previously observed correlation between HPV positivity and sexual activity frequency was not significant after correction. Sexual activity frequency showed significant negative correlations with number of sexual partners (r=-0.155, p < 0.01) and education level (r=-0.117, p < 0.01). BMI was also negatively correlated with education level (r=-0.084, p < 0.05). These relationships are illustrated in **Figure 1**.

### 3.5 Association between HPV status and cervical pathology

HPV-positive women exhibited significantly higher rates of abnormal cervical pathology than HPV-negative women (p < 0.001). Among HPV-positive participants, 31.1% (41/132) were diagnosed with CIN2+ or squamous cell carcinoma, 18.9% (25/132) had CIN1, and 50.0% (66/132) had chronic cervicitis. In contrast, among HPV-negative women, 98.8% (502/508) had chronic cervicitis and only 1.2% (6/508) had CIN1; no cases of CIN2+ or squamous cell carcinoma were observed. These findings are presented in **Table 4**.

**Table 4:** Distribution of cervical pathology by HPV status (n=640).

Pathology diagnosis	HPV-positive (n=132) n (%)	HPV-negative (n=508) n (%)	χ²-value	p-value
Chronic cervicitis	66 (50.0)	502 (98.8)	218.74	<0.001
CIN1	25 (18.9)	6 (1.2)	87.32	<0.001
CIN2+	24 (18.2)	0 (0)	95.41	<0.001
Squamous cell carcinoma (SCC)	17 (12.9)	0 (0)	68.55	<0.001

## 4 Discussion

This study represents one of the largest analyses of HPV epidemiology among women aged ≥50 years in Xinjiang and provides novel insights into infection prevalence, genotype distribution, risk factors, and associations with cervical pathology.

### 4.1 HPV prevalence and genotype distribution

The overall HPV prevalence of 20.6% observed in this cohort is higher than the 6.75% previously reported in a study conducted in southern Xinjiang (19), but remains consistent with global meta-analyses indicating that HPV prevalence among women aged >50 years typically ranges from 10% to 25% (20). This moderately elevated prevalence may be attributable to several factors, including the larger and more representative sample size of the present study and the inclusion of a substantial proportion of women from rural areas (38.5% of the sample), where access to regular and effective cervical cancer screening is often limited (21). In addition, demographic and immunological characteristics of older women—such as prolonged lifetime exposure to HPV, increased likelihood of persistent infection, and age-related decline in immune competence—may collectively contribute to the observed prevalence.

With respect to genotype distribution, HPV16 was the most prevalent high-risk genotype, detected in 29.5% of HPV-positive women. This finding is consistent with global and regional evidence underscoring the dominant role of HPV16 in the pathogenesis of invasive cervical cancer, accounting for approximately 50%–60% of cases worldwide (22). Notably, HPV53 was the second most common genotype (18.2%), a frequency higher than that reported in many other regions of China. Although HPV53 is classified as a high-risk type, its oncogenic potential is generally considered lower than that of HPV16 and HPV18. Its relatively high prevalence in this cohort may reflect regional variation in viral phylogenetics and host susceptibility, potentially influenced by the unique ethnic composition of Xinjiang. Previous studies have reported relatively high frequencies of HPV53 in certain Central Asian and European populations (23, 24), suggesting that geographic and ethnic factors may contribute to genotype distribution patterns. In addition, HPV58—another well-established high-risk genotype—was identified in 12.1% of HPV-positive cases, reinforcing its recognized importance in East Asian populations (25). Together, these findings highlight the importance of incorporating regionally prevalent HPV genotypes into vaccination strategies and screening algorithms to improve preventive effectiveness.

### 4.2 Risk factors for HPV positivity

The most notable finding regarding risk factors was the strong inverse association between a history of cervical surgery (including conization, LEEP, or cervical biopsy) and HPV positivity (OR=0.015). This result warrants cautious interpretation. Given the cross-sectional design, the observed association almost certainly reflects confounding by indication, also referred to as treatment selection bias (26). Furthermore, this pattern is consistent with reverse causality: the surgical intervention is a consequence of prior HPV detection and treatment, not a cause of reduced infection risk. Women undergo cervical surgical procedures precisely because they have been diagnosed with, or are suspected of having, HPV-related cervical abnormalities. Successful excision of the transformation zone may result in viral clearance, leading to negative HPV test results during subsequent follow-up. Consequently, women with a history of cervical surgery in this study are likely those who were effectively treated for prior HPV infection. Accordingly, this finding should not be interpreted as evidence that cervical surgery prevents new HPV infections, but rather as an indicator of successful treatment and clearance of previous infection. This interpretation is further supported by the ordinal logistic regression analysis, which demonstrated that a history of cervical surgery was associated with significantly lower odds of advanced cervical pathology at the time of study enrollment (27).

In contrast, analyses of sexual behavior yielded inconsistent findings. Although univariate analysis suggested higher sexual activity frequency among HPV-positive women, this association did not persist in the multivariate model or in correlation analyses after correction of a prior coding error (28). As such, no definitive conclusions can be drawn regarding the relationship between sexual activity frequency and HPV status in this cohort. This inconsistency may be attributable to limitations inherent in retrospective collection of sensitive behavioral data, potential recall bias, and the relatively limited variability in sexual behavior among older women (29, 30). Other behavioral factors, including smoking and alcohol consumption, were reported by only 4.1% of participants, consistent with the generally low prevalence of these behaviors among older Chinese women (31–33). Although smoking and alcohol use are established risk factors for HPV persistence and disease progression in younger populations (34, 35), their low frequency in this cohort likely limited statistical power to detect meaningful associations.

### 4.3 HPV and cervical pathology

The results demonstrate a strong correlation between HPV infection and histopathological abnormalities of the cervix. Among HPV-positive women, 31.1% were diagnosed with high-grade lesions (CIN2+) or invasive squamous cell carcinoma, compared with no cases of CIN2+or squamous cell carcinoma in the HPV negative group. This striking disparity underscores the central role of hrHPV infection in cervical carcinogenesis and highlights the potential severity of infection outcomes in older women, who are more likely to harbor persistent infections that have progressed to advanced stages. It is particularly concerning that 12.9% of HPV-positive women already had invasive cancer at the time of diagnosis, emphasizing the critical need for earlier and more inclusive screening in this age group.

These findings are consistent with previous studies indicating that the risk of cervical cancer increases with age, particularly in populations with limited access to regular screening. The high incidence of advanced disease among older women suggests that many have not undergone adequate or timely screening, allowing precancerous lesions to progress to invasive carcinoma over time. Therefore, extending organized screening programs to include women beyond 50 years of age should be considered a public health priority in Xinjiang and similar settings.

### 4.4 Potential mechanisms and contextualization

The inverse association between cervical surgery history and HPV positivity can be mechanistically explained by the therapeutic aim of these procedures: removal of the cervical transformation zone, the primary site of HPV persistence and neoplastic progression (36). Excision eliminates cells harboring integrated HPV DNA, thereby achieving virological clearance in a substantial proportion of cases (37). Subsequent negative HPV test results in these women therefore reflect successful treatment outcomes rather than altered susceptibility to infection.

The unusually high prevalence of HPV53 observed in this study also warrants further investigation. Xinjiang’s unique ethnic composition, including substantial Uygur, Kazakh, and other minority populations, may be associated with host genetic variation affecting immune responses to specific HPV genotypes (38–41). Phylogenetic analyses comparing HPV53 variants from Xinjiang with those from other regions could help determine whether region-specific viral subtypes exhibit distinct biological behaviors.

Finally, the extremely low HPV vaccination rate (0.2%) observed in this cohort is concerning but expected, given that current national immunization programs in China primarily target adolescents and young women. Although the prophylactic efficacy of HPV vaccination is greatest when administered before sexual debut, emerging evidence suggests that vaccination may still provide benefits to older women by preventing new infections and potentially facilitating clearance of existing infections. In regions with high HPV prevalence such as Xinjiang, expanding vaccination strategies to include mid-adult and older women may therefore represent a valuable complement to screening efforts.

### 4.5 Limitations

Several limitations of this study should be acknowledged. First, the retrospective design introduces the possibility of selection bias (42), as the study included only women who sought gynecological care at a tertiary hospital. This may have resulted in overrepresentation of symptomatic women or those with abnormal screening results, thereby limiting generalizability to the broader population of older women in Xinjiang. Second, owing to the retrospective nature of data collection (43), important potential confounders—including detailed sexual history, partner infection status, condom use, and immunological parameters—were unavailable. Third, confounding by indication represents a major limitation for the variable “history of cervical surgery.” The strong inverse association observed (OR=0.015) likely reflects successful prior treatment rather than a true protective effect against incident HPV infection. This cross-sectional analysis cannot disentangle treatment effects from prevention. Fourth, viral load and HPV integration status were not assessed, although both are important predictors of persistence and disease progression. Similarly, the absence of biomarkers such as p16/Ki67 immunohistochemistry (44) limited confirmation of the oncogenic activity of detected HPV infections. Fifth, although distribution normality was assessed and statistical tests were selected accordingly (**Supplementary Table S3**; Methods), retrospective collection of sensitive behavioral variables (e.g., sexual activity) remains subject to recall bias, potentially affecting measurement accuracy. Sixth, although *post hoc* power analysis confirmed adequate power for the primary association, the sample size may still have been insufficient to detect weaker associations, particularly for low-prevalence exposures such as smoking. Seventh, despite the ethnic diversity of Xinjiang, analyses were not stratified by ethnicity because ethnic identifiers were incompletely recorded in medical records (45), limiting exploration of ethnic variation in HPV prevalence and genotype distribution. Finally, the cross-sectional design precludes assessment of causal relationships or long-term outcomes such as HPV persistence or regression. Prospective cohort studies with extended follow-up are needed to better characterize HPV natural history in older women.

### 4.6 Clinical implications

Based on our findings, we recommend three key strategies: (1) expand screening programs to include women aged ≥50 years in Xinjiang, particularly those without a history of cervical surgery; (2) develop genotype-specific prevention strategies, prioritizing HPV vaccines covering HPV16 and HPV53 for older women in high-risk regions; and (3) improve vaccination rates through targeted public health campaigns aimed at postmenopausal women, who are often overlooked in current immunization efforts (46). This third suggestion is intended as a research-informed public health consideration to address a protection gap, rather than a direct reflection of current national vaccination guidelines, which primarily target adolescents and young women. In addition, healthcare providers should be educated about the unique HPV risk profile of older women, particularly in resource-limited settings. Integrating HPV testing into routine gynecological care for menopausal women could facilitate earlier detection and reduce cervical cancer mortality in this underserved population.

## 5 Conclusion

In summary, this study identified a high HPV prevalence among older women in Xinjiang, dominated by genotypes HPV16 and HPV53. A history of cervical surgery was strongly associated with lower HPV detection, likely reflecting successful prior treatment rather than a protective effect against new infection. The association between sexual activity and HPV status was inconsistent. Collectively, these findings underscore the urgent need to extend screening programs and tailor prevention strategies for this underserved age group.

## Data Availability

The original contributions presented in the study are included in the article/**Supplementary Material**. Further inquiries can be directed to the corresponding author.
