# Supplementary material for: Human papillomavirus prevalence, genotype distribution, risk factors, and cervical pathology association in women aged 50 years and older: a retrospective cross-sectional study in Xinjiang, China
Source: Front Oncol. 2026 Jan 12;15:1694755. doi: 10.3389/fonc.2025.1694755 (PMC12832329; doi:10.3389/fonc.2025.1694755)
Supplement: Supplementary file 1 [file Table1.docx]

| **Supplementary Table S1. Univariate binary logistic regression analysis of factors associated with HPV positivity (n=640).** | | | | |
| --- | --- | --- | --- | --- |
| **Variable** | **Category / Unit** | **Odds Ratio (OR)** | **95% Confidence Interval (CI)** | **p-value** |
| **Age group** | 50-60 years (Ref) | 1.00 | – | – |
|  | 61-70 years | 1.12 | 0.68 – 1.83 | 0.657 |
|  | >70 years | 0.88 | 0.42 – 1.86 | 0.738 |
| **Education level** | Secondary or below (Ref) | 1.00 | – | – |
|  | Junior college | 0.78 | 0.36 – 1.70 | 0.529 |
|  | Bachelor's degree | 0.61 | 0.12 – 3.08 | 0.551 |
| **Residence** | Urban (Ref) | 1.00 | – | – |
|  | Rural | 1.15 | 0.78 – 1.69 | 0.485 |
| **Sexual activity frequency** | Per category increase* | 1.183 | 1.020 – 1.372 | **0.027** |
| **Number of lifetime sexual partners** | Per partner increase | 1.09 | 0.94 – 1.26 | 0.248 |
| **Smoking/Alcohol use** | No (Ref) | 1.00 | – | – |
|  | Yes | 1.45 | 0.62 – 3.37 | 0.394 |
| **Menopausal status** | No (Ref) | 1.00 | – | – |
|  | Yes | 1.32 | 0.46 – 3.80 | 0.604 |
| **Menopausal age (years)** | Per year increase | 0.95 | 0.84 – 1.08 | 0.424 |
| **Parity** | Per birth increase | 0.97 | 0.85 – 1.11 | 0.655 |
| **Cervical surgery history** | No (Ref) | 1.00 | – | – |
|  | Yes | 0.016 | 0.009 – 0.030 | **<0.001** |
| **BMI category** | Normal (18.5-24.9) (Ref) | 1.00 | – | – |
|  | Overweight (25.0-29.9) | 0.94 | 0.64 – 1.39 | 0.762 |
|  | Obese (≥30.0) | 0.82 | 0.41 – 1.64 | 0.571 |
| **BMI (continuous, kg/m²)** | Per unit increase | 0.99 | 0.95 – 1.03 | 0.621 |

**Note:** Ref = Reference category. Sexual activity frequency was coded ordinally (1=None, 2=2-3 times/6 months, 3=1-3 times/month, 4=≥1 time/week). Variables with p < 0.1 (highlighted in bold) were included in the multivariate logistic regression model presented in the main text.
