# Supplementary material for: Human papillomavirus prevalence, genotype distribution, risk factors, and cervical pathology association in women aged 50 years and older: a retrospective cross-sectional study in Xinjiang, China
Source: Front Oncol. 2026 Jan 12;15:1694755. doi: 10.3389/fonc.2025.1694755 (PMC12832329; doi:10.3389/fonc.2025.1694755)
Supplement: Supplementary file 2 [file Table2.docx]

| **Supplementary Table S2. Ordinal logistic regression analysis of factors associated with cervical pathological progression (n=640)** | | | | |
| --- | --- | --- | --- | --- |
| **Variable** | **Category / Unit** | **Proportional Odds Ratio (OR)** | **95% Confidence Interval (CI)** | **p-value** |
| **Cervical surgery history** | No (Ref) | 1.00 | – | – |
|  | Yes | 0.12 | 0.06 – 0.24 | **<0.001** |
| **HPV status** | Negative (Ref) | 1.00 | – | – |
|  | Positive | 152.80 | 86.50 – 270.00 | **<0.001** |
| **Age group** | 50-60 years (Ref) | 1.00 | – | – |
|  | 61-70 years | 1.08 | 0.65 – 1.80 | 0.761 |
|  | >70 years | 1.05 | 0.48 – 2.31 | 0.903 |
| **Sexual activity frequency** | Per category increase | 1.04 | 0.86 – 1.25 | 0.692 |
| **BMI category** | Normal (Ref) | 1.00 | – | – |
|  | Overweight | 1.01 | 0.70 – 1.45 | 0.968 |
|  | Obese | 0.93 | 0.47 – 1.84 | 0.831 |
| **Education level** | Secondary or below (Ref) | 1.00 | – | – |
|  | Junior college | 0.85 | 0.40 – 1.81 | 0.674 |
|  | Bachelor's degree | 0.70 | 0.14 – 3.49 | 0.661 |
| **Parity** | Per birth increase | 0.98 | 0.86 – 1.12 | 0.780 |

**Model Fit Statistics:**

- **Test of Parallel Lines (Proportional Odds Assumption):** χ² = 9.15, df = 14, p = 0.822
- **Model Chi-Square:** χ² = 412.73, df = 12, p < 0.001
- **Nagelkerke Pseudo R²:** 0.68
- **-2 Log Likelihood:** 345.22

**Note:** Ref = Reference category. The proportional odds assumption was not violated (p > 0.05). The model shows that **cervical surgery history** is strongly associated with a lower odds of having more severe cervical pathology, and **HPV positivity** is overwhelmingly associated with higher odds of severe pathology, consistent with the main findings. Other covariates did not show significant independent associations.
