# Supplementary material for: Human papillomavirus prevalence, genotype distribution, risk factors, and cervical pathology association in women aged 50 years and older: a retrospective cross-sectional study in Xinjiang, China
Source: Front Oncol. 2026 Jan 12;15:1694755. doi: 10.3389/fonc.2025.1694755 (PMC12832329; doi:10.3389/fonc.2025.1694755)
Supplement: Supplementary file 3 [file Table3.docx]

| **Supplementary Table S3. Assessment of Normality for Continuous Variables (n=640)** | | | | |
| --- | --- | --- | --- | --- |
| **Continuous Variable** | **Shapiro-Wilk Statistic (W)** | **p-value** | **Distribution Judgment** | **Descriptive Statistic Used** |
| **Age (years)** | 0.992 | 0.064 | Approximately Normal | Mean ± SD: 56.8 ± 7.2 |
| **Menopausal Age (years)** | 0.981 | **0.012** | Non-Normal | Median (IQR): 49 (48, 51) |
| **BMI (kg/m²)** | 0.987 | **0.043** | Non-Normal* | Median (IQR): 25.8 (23.1, 28.5) |
| **Parity (number)** | 0.941 | **<0.001** | Non-Normal | Median (IQR): 2 (2, 3) |
| *Note: BMI was treated as a categorical variable in primary analyses. For correlation analysis (Section 3.4), a square-root transformation was applied to approximate normality. | | | | |
